# Burden of acute gastroenteritis among children younger than 5 years of age – a survey among parents in the United Arab Emirates

**DOI:** 10.1186/1471-2431-12-74

**Published:** 2012-06-18

**Authors:** Mohammad Howidi, Nawal Al Kaabi, Antoine C El Khoury, Agnes Brandtmüller, Laszlo Nagy, Etienne Richer, Wissam Haddadin, Mohamad S Miqdady

**Affiliations:** 1 Pediatrics department, Mafraq Hospital, Abu Dhabi, UAE; 2 Pediatric Infectious Diseases, Sheikh Khalifa Medical City, Abu Dhabi, UAE; 3 Global Health Outcomes, Merck & Co., Inc, WP97-A217, Sumneytown Pike, West Point, PA, 19486-0004, USA; 4 Budapest, Hungary; 5 MSD Hungary, Budapest, Hungary; 6 Biomedcom Consultants inc, Montreal, QC, Canada; 7 Merck Sharp and Dohme (MSD), Abu Dhabi, PO Box 29034, UAE; 8 Pediatric Gastroenterology, Sheikh Khalifa Medical City, Abu Dhabi, UAE

## Abstract

**Background:**

Despite its high incidence among children under the age of five, little is known about the burden of pediatric gastroenteritis outside the medical setting. The objective of this study was to describe the burden of acute gastroenteritis among children residing in the United Arab Emirates, including those not receiving medical care.

**Methods:**

A quantitative cross-sectional survey of 500 parents of children under 5 years of age who had suffered from acute gastroenteritis the preceding three months was conducted in the cities of Abu Dhabi and Al Ain. Data collected included respondent characteristics, disease symptoms, medical care sought, and parental expenditures and work loss. Data were analyzed using parametric and non-parametric statistical methods.

**Results:**

Vomiting and diarrhea episodes lasted on average between 3 and 4 days. Overall, 87% of parents sought medical care for their children; 10% of these cases required hospitalization with an average length of stay of 2.6 days. When medical care was sought, the average parental cost per gastroenteritis episode was US$64, 4.5 times higher than with home care only (US$14). Nearly 60% of this difference was attributable to co-payments and medication use: 69% of children used oral rehydration solution, 68% antiemetics, 65% antibiotics and 64% antidiarrheals. Overall, 38 parents missed work per 100 gastroenteritis episodes for an average of 1.4 days.

**Conclusions:**

Given its high incidence, pediatric gastroenteritis has an important financial and productivity impact on parents in the United Arab Emirates. To reduce this impact, efforts should be made both to prevent acute gastroenteritis and to optimize its treatment.

## Background

Annually about two billion cases of diarrheal diseases occur among children under the age of five globally [1]. Despite the fact that acute gastroenteritis can be prevented the disease still affects children, particularly under the age of two [1]. Gastroenteritis results from an inflammation of the gastrointestinal tract commonly caused by viral pathogens and less frequently by bacterial or parasitic organisms [2,3]. Every year about 1.5 million children die from diarrheal diseases, mostly in developing countries; this makes diarrheal diseases the second most common cause of death among children under the age of five following pneumonia [1].

Although the burden of diarrheal diseases among children under the age of five is heavy, improved prevention is achievable. Personal and food hygiene, including the use of clean water sources, are key measures to prevent transmission of these diseases [1]. Breastfeeding, especially under 6 months of age, also effectively protects infants [1]. Rotavirus vaccination has been widely available for children since 2006 [4] and is now recommended worldwide [1,3]. A growing body of evidence now supports the benefits of this vaccination and its universal implementation, both in the developed [5] and developing world [6].

Treatment can also be improved, in part through better adherence to recent guidelines [3]. The most important complication of gastroenteritis and the leading cause of death is dehydration; the mainstay treatment for gastroenteritis is oral rehydration [3]. Antiemetics are another treatment option that, despite sporadic use as adjuvant therapy, is not formally recommended in any international guidelines [3]. Finally, two other treatment options, antidiarrheals and routine antibiotics, are not recommended for pediatric gastroenteritis infections [3].

Rotavirus is the leading cause of severe gastroenteritis in the pediatric population worldwide [4]. These infections mainly occur in children between 6 and 24 months of age [7-9] and the age distribution in the United Arab Emirates (UAE) is close to what is reported worldwide [10,11]. By the age of five, most children will have had at least one episode of rotavirus infection [12,13]. The incidence of rotavirus gastroenteritis (RVGE) is independent of socio-economic status, although deaths mainly occur in developing countries due to limited access to healthcare in these countries [6,8].

The burden of RVGE in the Middle-East was recently reviewed [14]. Some broad regional estimates can be made, keeping in mind regional heterogeneity (socio-economical, geographical, environmental, cultural) and methodological differences. Rotavirus was detected in 16 (Saudi Arabia) to 61 (Syria)% of all gastroenteritis stool samples analyzed, and gastroenteritis hospitalization proportions ranged from 14 to 57% for RVGE compared to 14 to 28% for non-RVGE. Annual mortality rates due to RVGE vary widely from less than 10 per 100,000 (Bahrain, Israel, Kuwait, Oman, Qatar, UAE) to over 100 per 100,000 of population under five years of age (Yemen, Iraq). Although available data were limited, estimated RVGE-related direct medical costs per episode ranged from US$467 in Oman to US$1117 in Israel (2008 US$) [14].

Most studies on the burden of RVGE conducted so far have focused on hospitalized patients or those seeking medical care, but there is a knowledge gap on the burden of gastroenteritis outside the medical setting [14]. Moreover, very few studies addressed gastroenteritis and rotavirus infection in the UAE [10,11]. Hence the goal of this study was to describe the burden of acute gastroenteritis among children residing in the UAE, including those for whom parents did not seek medical care.

## Methods

### Design and setting

A quantitative cross-sectional survey was conducted through face to face interviews in the Emirate of Abu Dhabi; cities of Abu Dhabi (66% of the respondents) and Al Ain (34% of the respondents) from March 13 to April 20, 2010. The study was approved by the ethics committee of the Sheikh Khalifa Medical City, Abu Dhabi, UAE.

### Participants

Participants were recruited randomly in outdoor places frequented by parents of young children (e.g. nurseries and schools’ parkings, public parks, streets etc.) and through referrals whereby additional potentially eligible respondents were identified among initial respondents’ contacts.

A recruitment form was used to confirm eligibility of the respondents. Eligible respondents were parents or caregivers of children less than five years of age who had suffered from acute gastroenteritis (diarrhea or vomiting) in the preceding three months. A total of 1184 parents of children below five years of age were identified; of these, 500 eligible respondents were selected, 100 per each year of children’s age between birth and the fifth birthday, based on the child who had the most recent episode in the household within the last three months.

### Data collection

The main questionnaire was designed to gather information about the last episode of gastroenteritis occurring in any of the respondent children below five years of age. Respondents also completed a consent form. Cost data were collected and are presented as 2010 United Arab Emirates dirham (AED). Cost data were converted to 2010 US dollars (US$) using the August 23, 2010 exchange rate. Data were collected on respondent characteristics, disease symptoms, medical care sought, economic burden and parental work loss.

### Statistical analysis

Mean responses were calculated for children within each of the five 1-year age brackets. Overall means for the whole study population of children were also calculated by averaging the means for the five age groups, based on the assumption that the number of children in the source population (i.e., the total population of 0- to 4-year olds in the UAE) is approximately equal within each 1-year age bracket. Statistical analyses were performed using GraphPad Prism v5.04. One-way analysis of variance (ANOVA) with Tukey-Kramer multiple comparison test (to compare all pairs of age groups) were used to determine if the mean average duration of episodes of disease was different between the age groups. When Gaussian distribution could not be assumed (average length of stay, average cost per age group for non-medical expenses and days of work missed), the non-parametric Kruskal-Wallis test with Dunn’s multiple comparison test were used to compare the means. Two-way ANOVA was performed using the average parental expenses to consider the overall combine effect of transportation, co-payments, self-paid medications and non-medical care. Two-tailed t test were performed to compare average non-medical expenses between those seeking and those not seeking medical care.

## Results

### Respondent characteristics

Socio-demographic characteristics of the respondents are presented in Table 1 along with the corresponding data for the UAE population, as available. The surveyed population encompassed all UAE ethnicities; UAE locals represented 22%, Arab expatriates 36%, Asians 40% and Westerners 3%. In comparison, according to the 2005 census, 38% of the UAE population aged 0 to 4 years consisted of nationals, but only 20% of the overall UAE population [15]. Due to massive immigration, by 2010 the share of nationals in the overall UAD population was estimated to have declined to 11% [16]. (No data on the population distribution by age were available for 2010.) The survey sample was residing in the two fourth and fifth largest cities of the UAE, home to approximately 24% of the UAE population [17]. Fewer than 23%of the families surveyed had more than one child less than five years of age (Table 1). Additionally and as expected, the disease was not restricted to the lower socio-economic classes, over 60% of the respondents reported monthly household income exceeding AED10,000 (US$2723, Table 1). In comparison, the mean monthly income was approximately AED18,250 (US$4900) in 2009 [18]. Sixty-four percent (64%, fathers and mothers combined) of the survey parents were university graduates or post-graduates, a proportion more than double as high as among the overall UAE population aged 10 years and older (26.6%) [19].

**Table 1:** Respondents’ profiles (N = 500) compared to the population of the UAE

Characteristics	N (%)	Population of UAE	Sources
UAE locals	111 (22.2)	11% - whole population estimate (2010) 38% among 0- to 4-year-old children (2005 census)	NBS [15,16]
Arab expatriates	180 (36.0)	23% Arab or Iranians, 50% South Asians, 8% Westerners and East Asians (1982)	World Factbook [20]
Asians	194 (38.8)		
Westerners	15 (3.0)		
Abu Dhabi	330 (66.0)	15.3%	City Population.de [17]
Al Ain	170 (34.0)	8.5%	City Population.de [17]
Percent urban	500 (100%)	> 80%	City Population.de [17]
1	387 (77.4)	NA	
2	105 (21.0)	NA	
3	7 (1.5)	NA	
4	1 (0.5)	NA	
< 10,000	199 (39.8)	Mean: 18,249 (2009)	Bundhun, 2009 [18]
≥ 10,000 to < 20,000	202 (40.4)		
≥ 20,000	99 (19.8)		
Post-graduate	10.2/3.9	3.8†	NBS [19]
University graduate	62.0/52.0	21.1†	NBS [19]
Technical/vocational training/diploma	10.8/10.6	5.5†	NBS [19]
Secondary school (12 years)	13.6/25.3	23.1†	NBS [19]
Intermediate school (9 years)	2.0/5.1	14.9†	NBS [19]
Primary school or less (6 years)	1.2/1.0	13.9†	NBS [19]
No formal school	0.2/2.2	18.6†	NBS [19]

* Data shown for 500 mothers and 498 fathers.

†Population aged 10 years and older (2009).

AED: United Arab Emirates dirham; NA: not available; NBS: National Bureau of Statistics of the United Arab Emirates; UAE: United Arab Emirates.

### Gastroenteritis characteristics

The 500 respondents had 623 children under the age of 5 years, 583 of whom had at least one gastroenteritis episode within the 12 months preceding the interview. Transmission between siblings had occurred only in 2 to 4% of the episodes (data not shown). Seventy-five parents (15%) reported having been told that their children had RVGE. Of the 583 children, the 500 with the most recent episode within the last three months were considered for the remainder of this study. Among these, the vomiting and diarrhea episodes generally lasted between 3 and 4 days, rarely extending beyond 10 days (Table 2). The mean duration of the episodes differed between the age groups (*P* = .0075), the main, and statistically significant, difference was observed between children of 1 to 2 and 2 to 3 years of age with mean (SD) durations of 4.1 (4.2) and 3.0 (1.9) days, respectively (*P* < .05).

**Table 2:** Duration (days, SD) of vomiting and diarrhea episodes by children’s age

	Age of child (years)					
	0 to < 1	≥ 1 to < 2	≥ 2 to < 3	≥ 3 to < 4	≥ 4 to < 5	0 to <5
	(N = 100)	(N = 100)	(N = 100)	(N = 100)	(N = 100)	(N = 500)
Average*	3.2 (2.1)	4.1 (4.2)†	3.0 (1.9)†	3.2 (2.0)	4.0 (2.6)	3.5 (2.7)
Minimum	1	1	1	1	1	1
Maximum	10	35	10	10	9	35

SD: standard deviation.

*ANOVA *P* = .0075.

†Tukey-Kramer multiple comparison test ≥ 1– < 2 vs ≥ 2– < 3: *P* < .05.

### Medical care

During the gastroenteritis episodes, 85 to 91% of the parents (depending on children’s age) sought medical care for their children, 60% of them consulting a pediatrician. Although medical consultation in various out-patient settings (pediatrician, primary health care physician, family doctor, pharmacy) was frequent, hospitalization was reported for only 7.1 to 11.6% (mean of 10.3%) of those who sought medical care and for 6.0 to 10.0% of all patients (mean of 9.0%), depending on age (Table 3). This hospitalization proportion tended to be higher for younger children, ranging from 12% in children below age of 3 years to 7% in those 4 to 5 years of age, while the peak average length of stay was reported among children of 1 to 3 years of age (3.5 to 3.7 days, Table 3, *P* = .03). Younger age also affected the number of visits to the emergency department, as parents of children below one year of age sought medical care there more often (average of 2.6 visits, Table 3).

**Table 3:** Medical care related to gastroenteritis

	Age of child (years)					
	0 to < 1	≥ 1 to < 2	≥ 2 to < 3	≥ 3 to < 4	≥ 4 to < 5	0 to <5
	(N = 100)	(N = 100)	(N = 100)	(N = 100)	(N = 100)	(N = 500)
Number (%) seeking medical care	86 (86)	87 (87)	87 (87)	91 (91)	85 (85)	436 (87)
Pediatrician	69 (1.5)	56 (1.5)	54 (1.4)	60 (1.3)	59 (1.5)	60 (1.5)
Out-patient physician	20 (1.4)	30 (1.7)	28 (1.5)	27 (1.4)	30 (1. 3)	27 (1.5)
Family doctor	11 (0.9)	7 (1.3)	11 (1.1)	4 (1.0)	9 (1.14)	8 (1.1)
Emergency department	11 (2.6)	19 (1.4)	15 (1.0)	15 (1.1)	15 (1.0)	15 (1.3)
Pharmacy	8 (1.0)	6 (1.8)	6 (1.2)	8 (1.5)	6 (1.2)	7 (1.4)
Hospital admission						
%	10	10	10	9	6	9.0
% among those who sought medical care	11.6	11.5	11.5	9.9	7.1	10.3
Average length of stay (days, SD)*	1.8 (1.3)†	3.5 (1.4)†	3.7 (2.9)	1.6 (0.7)	2.0 (0.9)	2.6 (1.8)

*Kruskal-Wallis: *P* = .0331.

† Dunn's Multiple Comparison Test between 0– < 1 and ≥ 1– < 2: *P* < .1.

No statistically significant differences between the age groups other than those displayed were observed.

Among those who sought medical care, almost 80% reported using medication at home (Table 4). Oral rehydratation solution was the most used medicine (69% among those who sought medical care), closely followed by antiemetic medication (68%) (Table 4). Antibiotics and antidiarrheals were also widely used (in 65% and 64% of cases, respectively) (Table 4).

**Table 4:** Medication used at home among those who sought medical care

						
	0 to < 1	≥ 1 to < 2	≥ 2 to < 3	≥ 3 to < 4	≥ 4 to < 5	0 to <5
	(N = 86)	(N = 87)	(N = 87)	(N = 91)	(N = 85)	(N = 436)
Number using medication at home, N (%)	68 (79)	68 (78)	71 (82)	74 (81)	67 (79)	348 (80)
Antiemetics	70	70	69	70	62	67.8
Electrolyte solution	69	68	78	55	75	68.6
Antibiotics	64	65	60	75	59	65.0
Antidiarrheals	63	64	61	74	59	64.4

No statistically significant differences between the age groups were observed.

### Economic burden

**Figure 1:**
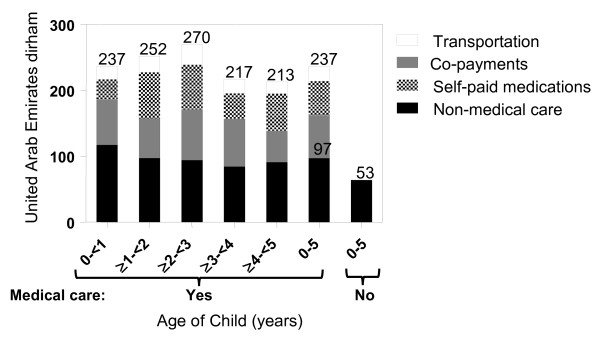
Average parental expenses per gastroenteritis episode among those seeking medical care.

Overall, the reported average cost per episode was 4.5 times higher when medical care was sought compared to home care alone with an average of AED237 (US$64) compared to AED53 (US$14) per episode. Nearly 60% of this difference was due to the cost of self-paid medications and copayments that were not existent for those not seeking medical care (Figure 1). Non medical care expenses, consisting of for example alternative diet, extra diapers, etc., were also higher among those seeking medical aid than for those not seeking medical aid with an average of AED97 (SD 134.5, US$26) compared to the AED53 (SD 50.83, *P* = .01, US$14 Figure 1). Non medical care expenses were also higher for younger age groups, declining from a mean of AED110 (US$30) in the 0 to 1 year age group to approximately AED85 (US$23) in children aged 2 to 5 (*P* = .0285), possibly reflecting the higher percentage of children using diapers below two years of age. Overall, total cost among those seeking medical care was highest in the 2 to 3 years age (AED270), mostly due to highest medication costs in this age group despite lower non-medical costs compared to younger age groups, although the differences was not statistically significant (Figure 1).

### Parental work loss

Work was missed by the parents during 32%of the pediatric gastroenteritis episodes (Table 5). Since some episodes caused both parents to miss work, an overall average of 38 parents missed work per 100 episodes (Table 5). On average 1.4 days of work were missed per episode and the age of the child affected the average days of work missed (*P* = .0097); the highest work loss (1.8) was observed for parents of the youngest children (Table 5). University graduates and post-graduates represented 78% of the respondents who reported missing work (70.1% university graduates and 8% post-graduates, not shown). In comparison, 64% of the overall surveyed proportion had a university diploma (Table 1).

**Table 5:** Parental work loss due to gastroenteritis in their children

	Age of child (years)					
	0 to < 1	≥ 1 to < 2	≥ 2 to < 3	≥ 3 to < 4	≥ 4 to < 5	0 to <5
	(N = 100)	(N = 100)	(N = 100)	(N = 100)	(N = 100)	(N = 500)
Proportion of episodes causing missed work, %	33	33	35	31	30	32.4
Number of parents missing work*	39	38	42	36	34	189
Average days of work missed (SD) †	1.8 (1.7)‡	1.5 (1.3)	1.1 (1.2)‡	1.5 (1.0)	1.1 (1.0)	1.4 (1.3)

*Note that in a number of cases both parents missed work due to the same episode.

† Kruskal-Wallis: *P* = .0097.

‡Dunn's Multiple Comparison Test between 0 to < 1 and ≥ 2 to < 3: *P* < .05.

No statistically significant differences between the age groups other than those displayed were observed.

## Discussion

In all age groups considered in this survey, the reported gastroenteritis episodes resulted in significant costs for parents and missed work days. A high proportion of parents sought medical care for their children (87%), mostly from pediatricians (60%). Given the incidence of gastroenteritis among young children and the proportion of hospitalization (10% among the 87% who sought medical care), gastroenteritis is likely to have had an important impact on the healthcare system, as recently reported in neighboring Oman for RVGE [21]. This high incidence also affects the overall costs, with an average cost per episode for parents seeking medical care 4.5 fold higher than for parents not seeking medical care. The impact on productivity is also important, as for every 100 episodes of gastroenteritis, 38 parents had to miss an average of 1.4 work days.

To our knowledge, this constitutes the first report on the burden of pediatric gastroenteritis and the costs to parents that includes children who did not receive medical attention in the UAE. Although a number of recent studies looked at the effect of RVGE on family life and parental work loss in the Middle East [22], the U.S [23], the E.U [24], and Asia [25], they were based on patients who sought medical care. A telephone survey pertaining to acute gastroenteritis was conducted in Ireland in 2003 and included those who did not seek medical attention; but this survey encompassed the general population (not focusing on children) and did not look at parental work loss caused by pediatric gastroenteritis [26]. Nevertheless, it is interesting to note that the frequency of medical care seeking for children below five years of age in Ireland was lower than in the present study, with only 48.6% being seen by a physician [26]. This lower percentage may have been affected by the case definition of gastroenteritis, as in the Irish survey diarrhea with at least three loose stools per day or bloody diarrhea or vomiting with other symptoms was defined as gastroenteritis [26], while in our study both vomiting and diarrhea were required for patients to be included.

As shown in a recent review, the proportion of gastroenteritis cases caused by rotavirus varies widely in the Middle-East, ranging from 16%in Saudi Arabia to 61%in Syria [14]. In the UAE, rotavirus has been reported in 21.4%of stool samples collected between 1990 and 1992 in a pediatric ward [10] and in 25% of the stool samples submitted for rotavirus detection in 2006 (79% of the positive samples were from children under 3 years of age) [11]. A similar proportion (22%) of rotavirus type A in hospital samples has been reported among pediatric patients in neighboring Saudi Arabia [14]. From that, we can estimate the proportion of gastroenteritis cases in the present study caused by rotavirus infection to be around 20 to 25%. This estimate, stemming mainly from hospital settings, may overestimate the actual proportion of rotavirus infection among all gastroenteritis cases because rotavirus infections are more severe and thus might be overrepresented in the hospital setting. That being said, because 87% of the parents sought medical care for their children, this bias may be limited. This approximation is also plausible in light of the number of diagnosed RVGE reported by the parents; 75 reported such a diagnosis among 583 children with gastroenteritis episodes within the last 12 months. Although this represents only 13% of the cases, the actual proportion is likely to be higher because testing for rotavirus is not routinely performed, is not available in private clinics, and parents may not recall diagnosis of rotavirus infection.

This approximation also corroborates the reported RVGE proportion among acute gastroenteritis cases outside of the Middle East among children under the age of five. This proportion was recently reviewed in Central and Eastern Europe (1999 to 2009), where it ranged from an average of 22.0%in the Czech Republic to an average of 55.3% in Russia [26], and in Western Europe (1999 to 2010), where it ranged from an average of 25.3%in Greece to 63.5%in Norway [27]. The reported RVGE proportion among children with acute gastroenteritis generally fell into these ranges in other regions of the world; 43.3% in Brazil [28,29], 45% in Asia, 40% in sub-Saharan Africa [30], and 27% in the US [31]. Of note, active surveillance tends to result in higher reported rotavirus prevalence among cases of pediatric acute gastroenteritis compared to other methodologies [31], which could at least partly explain the relatively low RVGE proportion reported in the UEA [10,11].

There was an important difference in the average cost per episode among those who sought medical care compared to those who did not. Parents might seek medical care for more severe cases of gastroenteritis, which could explain in part the higher cost of these episodes. A high proportion of additional costs came from medication copayments and the cost of self-paid medications. Increased adherence to international guidelines on gastroenteritis management could potentially decrease these costs as guidelines recommend oral rehydration solution as the mainstay treatment of pediatric gastroenteritis.

A high proportion of gastroenteritis episodes (32%) caused work loss across all socioeconomic classes, even though the study encompassed episodes for which medical care was not sought, indicating an overall important impact of pediatric gastroenteritis. Seventy-nine percent (79%) of the respondents who reported missing work had a university diploma. This proportion may be high given that among the UAE population over 10 years of age 24.9% hold a university diploma (nationals 13.5%, non-nationals 28.0% [19]). Certainly, a direct comparison is not applicable as the population above 10 years of age differs from the study population. Nonetheless, the high percentage of parents with university diplomas who missed work demonstrates the impact of gastroenteritis on the highly educated, mostly expatriate, workforce present in the urban setting of the study, although it may limit the generalizability of the results to the UAE population overall.

Another limitation of this study relates to the selection of the respondents, which took place in two cities in the emirate of Abu Dhabi, one of the seven emirates comprising the UAE. One should then be careful when extrapolating the results to other emirates or to rural settings. However, the Abu Dhabi emirate is the most populated with 34.1% of the 2005 census population [32], and the UAE population is highly urbanized, with more than 80% living in the six largest cities [17]. Also, the selection of the study setting makes it possible to compare the gathered data with two previously published studies from the UAE pertaining to enteric pathogens [11] and rotaviruses [10], both conducted in the city of Al Ain.

The overall representativeness of the study population is difficult to assess. Given the rapidly changing demographics of the UAE — with a population estimated to have doubled between 2005 and 2010 mainly due to the influx of workers [16] — census data from 2005 is of limited relevance for 2010. Nevertheless, it is reassuring that the proportion of the sample who were UAE nationals in this study (22%) lies between the 2005 census data for the 0- to 4-year-old population (38%) [15] and the 2010 estimate for the whole population (11%) [16], which one would expect as only a small proportion of the people recently entering to work in the UAE are likely to have families with young children.

A possible additional limitation pertains to the dates of the survey and the seasonality of rotavirus infections. In the Middle East (with the exception of Egypt) [14], Europe [12] and Asia [29], November to April is the peak season of rotavirus infections. This correlates with previously published data from the UEA gathered between January 1990 to December 1992, although the pattern was variable during those three years [10]. As this survey was conducted in March and April and included gastroenteritis events that occurred in the last three months, it is then plausible to assume that the peak RVGE season effects were captured. This allowed the observation of the burden of this disease, but one should be careful when extrapolating the results to other months (from May to November), as the RVGE proportion might be lower.

Finally, the absence of information about the etiologic agent responsible for the gastroenteritis reported in this survey limits the analysis of the relative impact of rotavirus infection. Without this information, we have to rely on data concerning the percentage of rotaviral infections gathered in 1990-1992 [11] and 2006 [10] and assume that it did not change significantly over the years. This assumption may need to be revised as RVGE incidence may have changed since the rotavirus vaccine became available in the UAE in 2006.

## Conclusions

Gastroenteritis below the age of five has an important impact on parents in the UAE, with more than one day of work missed per episode causing work loss (32.4%) and an average cost of AED237 (US$64) per episode among the majority (87%) who sought medical care for their children. Based on survey response and on published studies, rotavirus is estimated to be the etiologic agent in 20 to 25% of all cases of gastroenteritis identified in this study, highlighting the potential benefits of increased prevention, including vaccination, in reducing the clinical and economic burden of this disease.

## Competing interests

El Khoury was an employee of Merck when this study was conducted. Nagy and Haddadin are employed by Merck &Co., Inc. Brandtmüller has been contracted by Merck to participate in this study. Richer is employed by BioMedCom consultants inc, which has received consultancy fees from Merck & Co., inc. to participate in this study. Howidi, Al Kaabi and Miqdady declare that they have no competing interests.

## Authors’ contributions

MH, NAK, ACEK, AB, LN, WH and MSM conceived and designed the study. ACEK and WH acquired the data. All authors substantially contributed to the analysis and interpretation of the data. ER drafted the article and the other authors revised the article critically. All authors reviewed and approved the final version of the manuscript.

## Pre-publication history

The pre-publication history for this paper can be accessed here:

http://www.biomedcentral.com/1471-2431/12/74/prepub
